# Carbon Monoxide as an Electron Donor for the Biological Reduction of Sulphate

**DOI:** 10.1155/2010/319527

**Published:** 2010-06-14

**Authors:** Sofiya N. Parshina, Jan Sipma, Anne Meint Henstra, Alfons J. M. Stams

**Affiliations:** ^1^Laboratory of Microbiology of Anthropogenic Environments, Winogradsky Institute of Microbiology, Russian Academy of Sciences, 117312, prosp. 60 let Oktyabrya, 7, b.2, Moscow, Russia; ^2^Laboratory of Chemical and Environmental Engineering (LEQUIA), University of Girona, 17071 Girona, Spain; ^3^Laboratory of Microbiology, Wageningen University, 6703 HB, Wageningen, The Netherlands

## Abstract

Several strains of Gram-negative and Gram-positive sulphate-reducing bacteria (SRB) are able to use carbon monoxide (CO) as a carbon source and electron donor for biological sulphate reduction. These strains exhibit variable resistance to CO toxicity. The most resistant SRB can grow and use CO as an electron donor at concentrations up to 100%, whereas others are already severely inhibited at CO concentrations as low as 1-2%. Here, the utilization, inhibition characteristics, and enzymology of CO metabolism as well as the current state of genomics of CO-oxidizing SRB are reviewed. Carboxydotrophic sulphate-reducing bacteria can be applied for biological sulphate reduction with synthesis gas (a mixture of hydrogen and carbon monoxide) as an electron donor.

## 1. Introduction

Sulphate reducers are anaerobic microorganisms that are able to use sulphate as a terminal electron acceptor [1]. They are widespread in anoxic habitats [2] and can use numerous substrates as electron donor for growth. These include sugars [3, 4], amino acids [5, 6], hydrogen [7], and one-carbon compounds, such as methanol [8–11], carbon monoxide [12–14], and methanethiol [15]. Even alkanes [16–19], alkenes [20], and short-chain alkanes [21], as well as aniline [22], benzoate, phenol, aromatic hydrocarbons [23–25], and phosphite [26] are used as electron donor. 

Sulphate-reducing bacteria play an important role in biodesulfurization processes. Industries that use sulphuric acid, sulphate-rich feedstocks, or reduced sulphur compounds generate wastewaters rich in sulphate [27]. Sulphate is removed from wastewater by the combined activity of SRB that generate sulphide and the subsequent partial oxidation of sulphide to insoluble elemental sulphur by sulphide oxidizing bacteria [28]. Biotechnological applications of sulphate reduction further include SO_x_ abatement from the flue gas of coal fueled power plants [27] and treatment of sulphate-rich, heavy metal contaminated wastewaters. Heavy metals such as Cu, Zn, Cd, Pb, Ni, and Fe can be removed from waste streams by precipitation with biogenic sulfide. Because of differences in solubility of products, the metals can be selectively precipitated, which enables their recovery and reuse as demonstrated at full-scale for a zinc smelting plant [29]. 

If sulphate-rich wastewaters contain no or insufficient amounts of suitable electron and carbon donors for sulphate reduction, external addition becomes a prerequisite. Examples of such wastewaters are waste streams generated in galvanic processes, in the detoxification of metal-contaminated soils, in the mining of heavy metals and coal, and in flue gas desulphurization [30]. The selection of the most appropriate electron donor depends on its sulphate reduction efficiency, costs, and residual pollution. The use of simple organic compounds (e.g., ethanol or methanol) or H_2_/CO_2_ is highly preferred over complex wastes, for example, molasses, tannery effluents, solid waste materials, and manure [27]. Especially H_2_ is often used for biotechnological sulphate reduction. Van Houten et al. [31] demonstrated that with a mixture of H_2_/CO_2_ (80% : 20%), sulphate reduction rates as high as 30 g SO_4_^2−^ L^−1^ d^−1^ are achieved within 10 days of operation in gas lift bioreactors. Alternatively, synthesis gas or methane can be used; however, the sulphate reduction rates with methane are still extremely low. Synthesis gas, a mixture of H_2_, CO and CO_2_ and some other trace gases [32], has been considered a cheap alternative for high-purity H_2_. Synthesis gas can be used either directly or after enriching its H_2_ content by means of a water-gas shift reaction, that is, the catalytic reaction in which CO is forced to react with water yielding carbon dioxide and hydrogen [33]. In addition, residual electron donors or side products should be low or easily removed. Here, we review the growth of SRB with H_2_/CO mixtures and with pure CO.

## 2. CO in the Metabolism of SRB

### 2.1. CO as an Electron Donor for Pure Cultures of SRB

Some pure cultures of hydrogen-utilizing sulphate-reducing bacteria are able to use carbon monoxide as a carbon and energy source [34–36]. A few species of mesophilic sulphate-reducers can grow at low concentrations (4.5–20%) of CO. The most tested CO-utilizing SRB are species of genera *Desulfovibrio* and *Desulfotomaculum* (Table 1). The following reactions (Gibbs energy, ΔG°′), calculated according to [37] play a role during growth of SRB on CO:

(1) $$\begin{array}{cc} & 4\text{CO}+{{\text{SO}}_{4}^{}}^{2-}+4{\text{H}}_{2}\text{O}\to {{4\text{HCO}}_{3}^{}}^{-}+{\text{HS}}^{-}+{3\text{H}}^{+}\\ & \Delta {\text{G}}^{{}^{\circ}\prime }=-37.1\;\text{kJ}/\text{mol}\;\text{CO}, \end{array}$$

(2) $$\begin{array}{cc} & 4\text{CO}+4{\text{H}}_{2}\text{O}\to \;{\text{acetate}}^{-}+{{2\text{HCO}}_{3}^{}}^{-}+{3\text{H}}^{+}\\ & \Delta {\text{G}}^{{}^{\circ}\prime }=-28.2\;\text{kJ}/\text{mol}\;\text{CO}, \end{array}$$

(3) $$\begin{array}{cc} & \text{CO}+2{\text{H}}_{2}\text{O}\to {{\text{HCO}}_{3}^{}}^{-}+{\text{H}}_{2}+{\text{H}}^{+}\\ & \Delta {\text{G}}^{{}^{\circ}\prime }=\;-15\;\text{kJ}/\text{mol}\;\text{CO}, \end{array}$$

(4) $$\begin{array}{cc} & 4{\text{H}}_{2}+{{\text{SO}}_{4}^{}}^{2-}+{\text{H}}^{+}\to {\text{HS}}^{-}+4{\text{H}}_{2}\text{O}\\ & \Delta {\text{G}}^{{}^{\circ}\prime }=-45.2\;\text{kJ}/\text{mol}\;{\text{H}}_{2}, \end{array}$$

(5) $$\begin{array}{cc} & 4{\text{H}}_{2}+{{2\text{HCO}}_{3}^{}}^{-}+{\text{H}}^{+}\to {\text{acetate}}^{-}\;+4{\text{H}}_{2}\text{O}\\ & \Delta {\text{G}}^{{}^{\circ}\prime }=-36.4\;\text{kJ}/\text{mol}\;{\text{H}}_{2}. \end{array}$$

First information on CO oxidation by thermophilic *Desulfotomaculum* species was published in 1985 by Klemps et al. [8]. These moderately thermophilic *Desulfotomaculum* species could grow slowly with CO (4–20% v/v) as the only energy source. Four thermophilic sulphate-reducing bacteria: *Desulfotomaculum thermoacetoxidans *strain CAMZ, *Thermodesulfovibrio yellowstonii*, *Desulfotomaculum kuznetsovii*, and *Desulfotomaculum thermobenzoicum *subsp.* Thermosyntrophicum* were studied in pure culture and in coculture with the thermophilic carboxydotrophic hydrogenogenic bacterium *Carboxydothermus hydrogenoformans* [12]. In pure culture, *Dtm. kuznetsovii* and *Dtm. thermobenzoicum *subsp.* thermosyntrophicum* were able to grow with CO. In the presence of hydrogen and carbon dioxide, CO concentrations as high as 50–70% were utilized. The latter two SRB coupled CO oxidation to sulphate reduction without intermediate H_2_ production (reaction (1)), but a large part of the CO was converted to acetate (reaction (2)). When grown in coculture with the carboxydotrophic bacterium *C. hydrogenoformans* [44], *Dtm. kuznetsovii* and *Dtm. thermobenzoicum *subsp. *thermosyntrophicum* could grow with 100% CO (P_CO_ = 120 kPa) [12]. In that experiment, hydrogen was formed from CO by *C. hydrogenoformans* (reaction (3)) and subsequently the sulphate-reducers used the generated hydrogen as electron donor (reaction (4)).

*Dtm. carboxydivorans* strain CO-1-SRB^T^ was isolated from an anaerobic bioreactor treating wastewater from several paper mills [13]. The bacterium could grow with 100% CO in the gas phase. In the presence of sulphate, CO was converted to H_2_ and CO_2_ and the generated H_2_ was used for sulphate reduction. In the absence of sulphate, CO was completely converted to H_2_ and CO_2_. Phylogenetically it was placed between *Dtm. nigrificans* and *Desulfotomaculum* sp. RHT-3 [45–47]. *Desulfotomaculum *sp. RHT-3 could utilize up to 50% CO maximally [13], whereas *Dtm. nigrificans* could grow up to 20% CO maximally, as was found before by Klemps et al. [8]. Higher concentrations completely inhibited their growth [13]. 

*Archaeoglobus fulgidus* is a thermophilic sulphate reducing Archaeon able to grow on several organic substrates [42, 43]. Recently, the ability of *A. fulgidus* to grow with CO and to couple growth on CO with sulphate reduction was demonstrated [14]. Sulphate is reduced with CO as electron donor without intermediate H_2_ production, but with transient formate formation. Additionally *A. fulgidus* grows acetogenically with CO in the presence and absence of sulphate and sulphate reduction to sulphide is not inhibited by CO [14].

### 2.2. CO Toxicity to SRB

CO is a substrate, but also an inhibitor of sulphate-reducing bacteria. The inhibiting CO concentration ranges from 2% up to 70% for different SRB. It was found that *Desulfovibrio desulfuricans*, growing on lactate and sulphate, tolerated a CO concentration up to 20%, although such a CO concentration caused a reversible type of growth inhibition [40, 48] (Table 1). *Desulfovibrio africanus, Desulfovibrio baculatus,* and some *Desulfovibrio desulfuricans* strains grow with lactate in the presence of 20% or less of CO. Higher CO concentrations inhibited growth completely [40]. CO concentrations exceeding 20% (v/v) inhibited growth of *Desulfosporosinus orientis *completely [8]. Growth of *Dtm. thermoacetoxidans* and *T. yellowstonii* with pyruvate was completely inhibited at CO concentrations as low as 2% in the gas phase [12], whereas *Dtm. kuznetsovii* and *Dtm. thermobenzoicum* subsp. *thermosyntrophicum* were capable of growth in the presence of CO (Table 1). 

Synthesis gas was used as an electron donor of sulphate reduction in bioreactors [49–52]. These studies with mixed microbial communities encountered the same problems as with pure cultures. The major restriction of synthesis gas utilization is the presence of CO, which can range from 5 to over 50% [53] and its toxicity towards SRB [33]. Du Preez and Maree [50] reported a sulphate reduction rate of 2.4 g SO_4_^2−^ L^−1^ d^−1^ with pure CO. Van Houten et al. [51, 54] achieved a sulphate reduction rate in the range of 6–8 g SO_4_^2−^ L^−1^ d^−1^, with a feed gas containing maximally 20% CO. Van Houten et al. [51, 55] showed that CO exerted a toxic effect on the SRB present in the biomass at gas phase CO concentrations between 5 and 20% as the sulphate reduction rates were limited to 8 g SO_4_^2−^ L^−1^ d^−1^, whereas with H_2_/CO_2_ the maximum sulphate reduction rate achieved was 30 g SO_4_^2−^ L^−1^ d^−1^ [31]. In the presence of CO, a layered biomass structure developed with acetogenic bacteria located at the outside of the biofilm, and SRB located deeper inside the biofilm probably as a protection mechanism towards CO toxicity [51, 55].

### 2.3. Enzymes of CO Metabolism and Genomics of CO-Oxidizing SRB

Of all sulphate reducers, only *A. fulgidus* and* Dtm. carboxydivorans* were not inhibited by the highest tested P_CO_ of 200 and 124 kPa, respectively [14, 56]. Presence of a CO-dehydrogenase in a sulphate reducer does not necessarily mean that it is less sensitive towards CO. Cell extracts of *Dtm. thermoacetoxidans *revealed a CO-dehydrogenase activity [57] and the whole-genome sequence of *T. yellowstonii* contains a CO-dehydrogenase gene as part of an ACS/CODH cluster (Table 2). However, both bacteria are completely inhibited by less than 2% of CO in the gas phase [12]. It might be that the CO-dehydrogenase of *T. yellowstonii *is inactive, since no CO-oxidizing activity was found in cell extracts of this bacterium and it needs acetate as carbon source for growth with H_2_ or formate as electron donors [58].

In recent reviews devoted to CO-utilization, genomics and CO-sensing mechanisms of anaerobic, aerobic bacteria and Archaea, it was concluded that there is no single mechanism of CO-utilization and CO-inhibition [59–61]. 

The mechanism of CO-inhibition in particular of sulphate reducers is not completely understood as well. Hydrogenases play a key role in the metabolism of hydrogenotrophic sulphate reducers, and also in the hydrogen-cycling mechanism that was proposed for *Desulfovibrio spp.* growing on lactate [62, 63]. It has been shown that CO is a competitive inhibitor of [FeFe]-hydrogenases where it binds to iron in the active site [62, 63]. Interestingly, CO also serves, together with CN^−^, as a coordinating ligand in the active site of [FeFe]-hydrogenases [64–67]. 

Inhibition of hydrogenase therefore could potentially explain at least part of the sensitivity of some sulphate reducers towards CO. Other metallo-enzymes could similarly be inhibited by CO. In *Dsv. desulfuricans* strain B-1388, the effect of CO on metabolism was studied in more detail. In this bacterium, 5% CO caused “nonspecific stress” resulting in a lower growth rate, increased content of cytochrome c, increased content of reduced pyridine nucleotides, and a low ATP concentration [68–70]. Therefore, it has been suggested that the function of the CO-dehydrogenase in this organism is to detoxify CO [70]. It was shown that hydrogenases of *Clostridium pasteurianum* are inhibited by binding of CO to iron in the active site, but whether this is the sole mechanism of inhibition is not clear [71]. Recent interesting results in this field-CO-tolerant hydrogenases exist that exclude CO from the active site by means of a narrow tunnel that allows hydrogen to pass, but not CO [72]. Since CO binds readily to metals, such as iron, other metal bearing proteins may be similarly affected.

The genome sequence of *Dsv. desulfuricans* strain B-1388 has not been solved, but the genome of seven other *Desulfovibrio spp.* and of ten other dissimilatory sulphate reducers has been sequenced (Table 2). At least 25 more whole-genome sequencing projects on sulphate reducers are ongoing. Of the seven available *Desulfovibrio *genomes, only *Desulfovibrio piger* does not contain a CO-dehydrogenase gene homologue. The CO-dehydrogenase gene context appears conserved in *Desulfovibrio spp.* with *cooA* and *cooC* homologues, respectively, up- and down-stream of the *cooS*, the gene fragment encoding for CO-dehydrogenase. CooA is a CO-binding transcriptional regulator [74] and CooC a CO-dehydrogenase maturation protein [75, 76]. Interestingly, *cooA* seems only present in the genomes of *Desulfovibrio spp., *but in none of the other available genomes of sulphate reducers. Further, CO-dehydrogenase gene context reveals little information about its possible physiological role in the metabolism. A *hyd * [FeFe]-hydrogenase deletion mutant of *Desulfovibrio vulgaris* Hildenborough formed transient amounts of CO up to 6000 ppm as part of a fermentation burst. Likely its CO-dehydrogenase is first involved in the production and then in the consumption of CO by this mutant, with electron transfer possibly linked to a general electron pool [77]. For *Dsv. desulfuricans* B-1388, it was also shown that CO-dehydrogenase activity in cell extracts increases over time in batch cultures of cells grown with lactate and sulphate as substrates [35, 78]. In late growth stages, the degradation of cellular material, for example, porphyrins, may release CO and this results in an increased CO-dehydrogenase expression through the action of CooA.

In the remainder of fully sequenced genomes of sulphate reducers (Table 2) that contain one or more CO-dehydrogenase genes, the CO-dehydrogenase is either part of an ACS/CODH cluster or is located alone or together with unexpected genes. In this group of sulphate reducers, only *Archaeoglobus fulgidus* is shown to grow with CO as substrate [14]. *A. fulgidus* contains three CO-dehydrogenase genes, two of the type typically found in archaeal ACS/CODH complexes and one homologous to *cooS *found in bacteria. The archaeal type of CO-dehydrogenases is paired together only with the epsilon subunit of ACS/CODH in *A. fulgidus*. The beta, gamma, and delta subunits of ACS/CODH appear only once in the genome, separate from the alpha epsilon pairs. It has been speculated that both archaeal type CO-dehydrogenases are involved in the acetyl-CoA pathway and that oxidation of CO by CooS provides electrons for reduction of CO_2_ to formyl methanofuran and possibly in the reduction of sulphate [14]. Direct experimental evidence for these roles, however, is still lacking.

The presence of multiple CODH genes in a single genome suggests that multiple physiological roles exist for CO-dehydrogenase. Besides a role in the ACS/CODH complex, CO could be oxidized by CODH and serve as an electron donor for sulfate reduction or CO-dehydrogenase could serve to detoxify CO, but clear experimental evidence is lacking. Clearly, the different sulphate reducers respond differently to CO. While based on available whole genome sequences it appears that most sulphate reducers contain a CO-dehydrogenase gene, their sensitivity towards CO allows speculation that levels of CO that are encountered by these microorganisms in their natural habitat are limited. It seems that only few sulphate reducers are insensitive towards CO, although more may be found.

## 3. Synthesis Gas as an Electron Donor for Biotechnological Sulphate Reduction

According to a cost estimation concerning the use of ethanol and H_2_ as electron donors for sulphate reduction, ethanol turned out to be cheaper for small-scale installations (<5 kmol h^−1^), whereas H_2_ is cheaper for larger installations, assuming the use of a high-purity H_2_ [55]. Synthesis gas, produced by, for example, steam reforming of natural gas or thermal gasification of coal, oil, biomass, or other organic matter and widely available as by-product of coal burners is a cheap alternative for high-purity H_2_ [55]. Furthermore, operational costs could be greatly reduced in case of on-site production of synthesis gas from coal, thus minimizing transportation costs. 

Several mesophilic full-scale anaerobic sludges, when incubated at 55°C, revealed the capability of hydrogenogenic CO conversion, whereas direct acetate production was not observed [79]. As mentioned earlier, especially hydrogenogenic CO conversion holds a promise for the use of synthesis gas in biotechnological sulphate reduction, for example, in thermophilic flue gas desulfurization. Therefore, the utilization of CO as a sole external electron donor in thermophilic (55°C) sulphate reduction was investigated in lab-scale gas lift bioreactors [80–82]. These reactors were inoculated with mesophilic granular sludge, with a high hydrogenogenic activity on CO at 55°C, from which the hydrogenogenic sulphate reducer *Dtm. carboxydivorans* [13] was isolated. 

Thermophilic (50–55°C) sulphate reduction rates of up to 20 mmol L^−1^ d^−1^ were obtained at hydraulic retention times (HRTs) between 6 and 14 hours [80] showing the potential of sulphate reduction with CO as an external electron donor. However, due to predominant methanogenic H_2_ consumption, sulphate reduction rates were generally low. Similar competition effects at 55°C leading to methanogenesis have been reported with methanol [83] and H_2_/CO_2_ [84]. Fast growth rates of the methanogens (generation time of 4.5 hours) enabled them to recover from imposed pH and temperature shocks, and they consumed more than 90% of the CO-derived H_2_ [80]. Nevertheless, steep increases in sulphide production in periods with low methanogenic activity suggest that once methanogenesis is eliminated, sulphate reduction with CO-rich gas as electron donor has a great potential [80].

Operation at HRTs shorter than the generation time of the methanogens allowed stable sulphate reduction for prolonged periods, and up to 95% of the CO-derived H_2_ was used for sulphate reduction [82]. The achieved sulphate reduction rates of 17 mmol·L^−1^·d^−1^ were limited by the amount of CO supplied and its conversion efficiency (about 85%) at higher CO loads likely results from biomass limitation. Methane production, however, persisted when operating under these conditions and increasing the HRT by returning it to values >5.5 hours resulted in a dominance of methanogenesis over sulfate reduction [82]. 

Although the operation of a thermophilic CO fed gas lift reactor at extremely low HRTs [82] clearly demonstrated the potential of CO or synthesis gas as an electron donor for biotechnological sulphate reduction, the low biomass concentrations present limited the applicable sulphate loads and thus its practical application. For high-rate biotechnological sulphate reduction processes, biomass retention is a prerequisite, and thus elimination or efficient suppression of methanogenesis is essential. Simple changes in operation conditions, such as pH, temperature, or salinity were ineffective for steering the competition for H_2_ utilization towards sulphate reduction [80]. Suppression of thermophilic hydrogenogenic methanogenesis has been demonstrated with the use of chemical inhibitors [85] or pretreatment of the sludge prior to inoculation [86–88]. However, the use of chemical inhibitors represents a potential risk as they might be released into the environment. Furthermore, adaptation to the inhibitor might result in increased operational costs due to an increasing demand of chemical inhibitors to maintain the same level of inhibition. Weijma and coworkers [89] observed that the application of a continuous dosing of 2 g·L^−1^ 2-bromoethanesulfonate to a bioreactor was ineffective for the suppression of methanogenesis, as methanogenesis resumed after two days. In contrast, Sparling et al. [90] successfully inhibited methane formation in an H_2_ producing anaerobic digester by the addition of 1% v/v acetylene in the headspace. 

Heat or acid/base treatment does not inhibit methanogens during reactor operation as would be the case with chemical agents, but rather eliminates methanogens before reactor operation is initiated. Such a pretreatment poses no environmental risk, because no harmful chemicals will be released, and furthermore, such a pretreatment in principle needs to be performed only once; whereas, chemical inhibitors need to be applied constantly most likely at an increased demand. Nevertheless, for the successful suppression of methanogens their complete elimination is a prerequisite, especially fast growing thermophilic methanogens.

In incubations at 55°C with *Dtm. carboxydivorans*, sulphide production occurred at a P_CO_ exceeding 100 kPa [56]. However, *Dtm. carboxydivorans* has a pH-dependent sensitivity for sulphide inhibition, that is, 9 mM sulfide at pH 7.2 and 5 mM at pH 6.5 cause complete inhibition [56]. Thus, most likely not toxicity of CO, but of sulphide determines the sulphate reduction capacity of *Dtm. carboxydivorans*, as bulk liquid CO concentrations will likely be kept low due to biological conversion. Therefore, to develop a high-rate sulphate-reducing bioreactor employing *Dtm. carboxydivorans*, additional features to maintain the sulphide concentration below inhibitory levels are required. To maintain sulphide concentrations sufficiently low, operation at slightly elevated pH-values could be considered or application of an alkaline H_2_S absorber through which the recycle gas is led. The H_2_S absorbed from the gas phase could be fed to a second micro-aerobic biological reactor, preferably operated at high pH to minimize consumption of chemicals, in which the sulphide is partially oxidized to elemental sulphur [28]. Another attractive option is the use of H_2_S extractive membranes [91] placed inside the bioreactor mixed liquor, which could result in direct recovery of elemental sulphur when combining with a Fe^3+^-containing extraction solution. 

Successful application of CO-containing H_2_-rich synthesis gas in biodesulphurization requires not merely tolerance for the presence of CO, but also the use of CO within the synthesis gas or its conversion products for sulphate reduction as well. The conversion of CO to acetate, although a potential substrate for SRB, can be considered a disadvantage as acetate gives rise to elevated effluent COD concentrations and requires the presence of both hydrogenotrophic and acetotrophic SRB. For the maximal utilization of syngas as electron donor, the distinct dominating groups of SRB should be capable of outcompeting other microorganisms that use H_2_ and acetate. Mesophilic SRB have been shown capable of outcompeting hydrogenotrophic methanogens [31, 92]. The competition between SRB and methanogens for acetate appears less predictable despite the thermodynamic and kinetic advantages ascribed to SRB [27].

## 4. Summary and Perspectives

Presently, only about ten sulphate-reducing bacteria and one archaeon are able to use CO as an electron donor for sulphate reduction. Most CO-utilizing sulphate reducers are moderately thermophilic, Gram-positive bacteria. Gram-negative SRB of the *Desulfovibrio *genus are more sensitive to CO. Still very little is known about the dissimilation and assimilation of CO by sulphate-reducing bacteria and the toxic effect of CO on their metabolism. The genomes of some Gram-negative sulphate-reducing bacteria are available as well as that of *Archaeoglobus fulgidus.* The only genome sequence of *Desulfotomaculum *sp.*-Dtm. reducens* is available, but no genes involved in the CO metabolism are present. The upcoming genomic data will give us information about the presence of the enzymes of CO-utilization among SRB. With genomes from CO-metabolizing Gram-positive sulphate reducers, a comparative analysis can be made of the CO metabolism of Gram-negative and Gram-positive sulphate reducers. Furthermore, more detailed investigation of acetyl-CoA pathway enzymes and hydrogenases of carboxydotrophic SRB might give more insight to CO-utilization by SRB. 

The occurrence of thermophilic microorganisms capable of a high-rate hydrogenogenic CO conversion as well as sulfate reduction in the presence of high levels of CO enables the use of CO containing synthesis gas in biotechnological desulphurisation. Successful application of H_2_-rich synthesis gas in biodesulphurisation, without the need for prior purification, requires tolerance for the presence of CO. Furthermore, it would be most beneficial to the overall sulfate reduction process if CO within the synthesis gas is used for sulphate reduction as well. Thus, both toxicity and potential metabolic use of CO as an electron donor are important factors governing the utilization potential of CO-rich synthesis gas for biotechnological sulphate reduction. In that respect, the recent discovery of CO tolerant SRB, such as* Dtm. carboxydivorans*, indicates that CO-rich synthesis gas holds promise as a cheap alternative electron donor for biodesulphurisation. Nevertheless, in order to enable high-rate biotechnological sulphate reduction employing *Dtm. carboxydivorans*, special attention should be directed to the competition for the electron donor with methanogens as well as inhibitory effects of the hydrogen sulphide formed.

## Figures and Tables

**Table 1:** Tolerance and use of CO by tested sulphate reducers.

	*T* (°C)	CO + carbon sources besides CO	Products formed		References
			With sulphate	Without sulphate	
*Desulfovibrio vulgaris *str.* Madison *	37	≤4,5% (+1 mmol/l sodium acetate)	H_2_, CO_2_, H_2_S	No growth	[38]
*Desulfovibrio baarsii *2st14	37	1.5% (+ formate)	Alanine, Aspartate Glutamate, H_2_S	Not reported	[39]
*Desulfovibrio desulfuricans, Desulfovibrio baculatus, Desulfovibrio africanus*	30	≤20%+ lactate	H_2_, CO_2_, H_2_S	Not tested	[40]
*Desulfosporosinus orientis* (former *Desulfotomaculum orientis*)	35–37	≤20% (+1 mmol/l sodium acetate)	H_2_, CO_2_, H_2_S	No growth	[8]
*Desulfotomaculum nigrificans*	55	≤20% (+1mmol/l sodium acetate)	H_2_, CO_2_, H_2_S	No growth	[8]
*Desulfotomaculum thermobenzoicum *subsp. *thermosyntrophicum *	55	≤50–70%	Acetate, CO_2_, H_2_S	No growth	[12, 41]
*Desulfotomaculum kuznetsovii*	55–60	≤50%	Acetate, CO_2_, H_2_S	No growth	[10, 12]
*Desulfotomaculum *sp. RHT-3	55	≤50%	H_2_, CO_2_, H_2_S	No growth	[10, 13]
*Desulfotomaculum carboxydivorans* CO-1-SRB	55	100%	H_2_, CO_2_, H_2_S	H_2_, CO_2_	[13]
* Archaeoglobus fulgidus* VC 16	75–80	≤80%	Acetate, CO_2_, H_2_S, formate (transient)	CO_2_, acetate, formate (transient)	[14, 42, 43]

**Table 2:** CO-dehydrogenase genes in completed genome sequences of sulphate reducers.

Species	strain	NCBI RefSeq	locus	total	bacterial^1^	archaeal^1^	ACS/CODH^2^
*Archaeoglobus fulgidus*	DSM4304	NC_000917	AF1100, 1849, 2397	3	1	2	2
*Desulfovibrio vulgaris*	Hildenborough	NC_002937	DVU0298	1	1	0	0
*Desulfovibrio vulgaris*	DP4	NC_008751	Dvul_1133	1	1	0	0
*Desulfovibrio vulgaris*	Miyazaki F	NC_011769	DvMF_2233	1	1	0	0
*Desulfovibrio desulfuricans*	G20	NC_007519	Dde_3028	1	1	0	0
*Desulfovibrio desulfuricans*	ATCC27774	NC_011883	Ddes_0382	1	1	0	0
*Desulfovibrio salexigens*	DSM2638	NZ_ACCN	DesalDRAFT	1	1	0	0
*Desulfotomaculum reducens*	MI-1	NC_009253	Dred_0652	1	1	0	0
^3^ *Desulforudis audaxviator*	MP104C	NC_010424	Daud_0870, 0105	2	1	1	2
*Desulfococcus oleovorans*	Hxd3	NC_009943	Dole_1461, 3185	2	2	0	1
*Syntrophobacter fumaroxidans*	MPOB	NC_008554	Sfum_2566, 2875	2	2	0	1
*Desulfatibacillum alkenivorans*	AK-01	NC_011768	Dalk_0680, 2379	2	2	0	1
*Thermodesulfovibrio yellowstonii*	DSM11347	NC_011269	THEYE_A1470	1	1	0	1
*Desulfobacterium autotrophicum*	HRM2	^4^CP001087	HRM2_16670, 41010, 43430	3	3	0	1

*Caldivirga maquilingensis*	IC-167	NC_009954		0			
*Desulfovibrio piger*	ATCC29098	NZ_ABXU		0			
*Desulfotalea psychrophila*	LSv54	NC_006138		0			

^1^ Number of CO-dehydrogenase genes present in the genome that are homologous to the CO-dehydrogenases commonly found in bacterial ACS/CODH or in archaeal ACS/CODH.

^2^ Number of CO-dehydrogenase genes present in a gene context that suggest it is part of acetyl-CoA synthase/CO-dehydrogenase complex.

^3^ It is predicted that *candidatus Desulforudis audaxviator *is a sulphate reducer based upon genome sequence [73].

^4^ Genbank accession number for *Desulfobacterium autotrophicum.*

## References

[1] Postgate JR (1979). *The Sulphate-Reducing Bacteria*.

[2] Muyzer G, Stams AJM (2008). The ecology and biotechnology of sulphate-reducing bacteria. *Nature Reviews Microbiology*.

[3] Ollivier B, Cord-Ruwisch R, Hatchikian EC, Garcia JL (1988). Characterization of *Desulfovibrio fructosovorans* sp. nov. *Archives of Microbiology*.

[4] Sass A, Rutters H, Cypionka H, Sass H *Desulfobulbus mediterraneus* sp. nov., a sulphate-reducing bacterium growing on mono- and disaccharides. *Archives of Microbiology*.

[5] Baena S, Fardeau M-L, Labat M, Ollivier B, Garcia J-L, Patel BKC (1998). *Desulfovibrio aminophilus* sp. nov., a novel amino acid degrading and sulfate reducing bacterium from an anaerobic dairy wastewater lagoon. *Systematic and Applied Microbiology*.

[6] Stams AJM, Hansen TA, Skyring GW (1985). Utilization of amino acids as energy substrates by two marine *Desulfovibrio* strains. *FEMS Microbiology Ecology*.

[7] Widdel F, Hansen TA, Balows A, Trüper HG, Dworkin M, Harder W, Schleifer K-H (1992). The dissimilatory sulfate- and sulfurreducing bacteria. *The Prokaryotes*.

[8] Klemps R, Cypionka H, Widdel F, Pfennig N (1985). Growth with hydrogen, and further physiological characteristics of *Desulfotomaculum* species. *Archives of Microbiology*.

[9] Nanninga HJ, Gottschal JC (1987). Properties of *Desulfovibrio carbinolicus* sp. nov. and other sulfate-reducing bacteria isolated from an anaerobic-purification plant. *Applied and Environmental Microbiology*.

[10] Nazina TN, Ivanova AE, Kanchaveli LP, Rozanova EP (1988). A new sporeforming thermophilic methylotrophic sulfate-reducing bacterium, *Desulfotomaculum kuznetsovii* sp. nov.. *Mikrobiologia*.

[11] Nazina TN, Turova TP, Poltaraus AB (1999). Phylogenetic position and chemotaxonomic characteristics of the thermophilic sulfate-reducing bacterium *Desulfotomaculum kuznetsovii*. *Mikrobiologiya*.

[12] Parshina SN, Kijlstra S, Henstra AM, Sipma J, Plugge CM, Stams AJM (2005). Carbon monoxide conversion by thermophilic sulfate-reducing bacteria in pure culture and in co-culture with Carboxydothermus hydrogenoformans. *Applied Microbiology and Biotechnology*.

[13] Parshina SN, Sipma J, Nakashimada Y (2005). *Desulfotomaculum carboxydivorans* sp. nov., a novel sulfate-reducing bacterium capable of growth at 100% CO. *International Journal of Systematic and Evolutionary Microbiology*.

[14] Henstra AM, Dijkema C, Stams AJM (2007). *Archaeglobus fulgidus* couples CO oxidation to sulfate reduction and acetogenesis with transietnt formate accumulation. *Environmental Microbiology*.

[15] Tanimoto Y, Bak F (1994). Anaerobic degradation of methylmercaptan and dimethyl sulfide by newly isolated thermophilic sulfate-reducing bacteria. *Applied and Environmental Microbiology*.

[16] Aeckersberg F, Rainey FA, Widdel F (1998). Growth, natural relationships, cellular fatty acids and metabolic adaptation of sulfate-reducing bacteria that utilize long-chain alkanes under anoxic conditions. *Archives of Microbiology*.

[17] So CM, Young LY (1999). Isolation and characterization of a sulfate-reducing bacterium that anaerobically degrades alkanes. *Applied and Environmental Microbiology*.

[18] Davidova IA, Duncan KE, Choi OK, Suflita JM (2006). *Desulfoglaeba alkanexedens* gen. nov., sp. nov. an *n*-alkane-degrading, sulfate-reducing bacterium. *International Journal of Systematic and Evolutionary Microbiology*.

[19] Cravo-Laureau C, Matheron R, Cayol J-L, Joulian C, Hirschler-Réa A (2004). *Desulfatibacillum aliphaticivorans* gen. nov., sp. nov., an *n*-alkane- and *n*-alkene-degrading, sulfate-reducing bacterium. *International Journal of Systematic and Evolutionary Microbiology*.

[20] Grossi V, Cravo-Laureau C, Meóu A, Raphel D, Garzino F, Hirschler-Réa A (2007). Anaerobic 1-alkene metabolism by the alkane- and alkene-degrading sulfate reducer *Desulfatibacillum aliphaticivorans* strain CV2803T. *Applied and Environmental Microbiology*.

[21] Kniemeyer O, Musat F, Sievert SM (2007). Anaerobic oxidation of short-chain hydrocarbons by marine sulphate-reducing bacteria. *Nature*.

[22] Schnell S, Bak F, Pfennig N (1989). Anaerobic degradation of aniline and dihydroxybenzenes by newly isolated sulfate-reducing bacteria and description of *Desulfobacterium anilini*. *Archives of Microbiology*.

[23] Rabus R, Nordhaus R, Ludwig W, Widdel F (1993). Complete oxidation of toluene under strictly anoxic conditions by a new sulfate-reducing bacterium. *Applied and Environmental Microbiology*.

[24] Harms G, Zengler K, Rabus R (1999). Anaerobic oxidation of *o*-xylene, *m*-xylene, and homologous alkylbenzenes by new types of sulfate-reducing bacteria. *Applied and Environmental Microbiology*.

[25] Morasch B, Schink B, Tebbe CC, Meckenstock RU (2004). Degradation of *o*-xylene and *m*-xylene by a novel sulfate-reducer belonging to the genus *Desulfotomaculum*. *Archives of Microbiology*.

[26] Schink B, Friedrich M (2000). Phosphite oxidation by sulphate reduction. *Nature*.

[27] Lens PNL, Visser A, Janssen AJH, Hulshoff Pol LW, Lettinga G (1998). Biotechnological treatment of sulfate-rich wastewaters. *Critical Reviews in Environmental Science and Technology*.

[28] Janssen AJH, Dijkman H, Janssen G, Lens PNL, Hulshoff Pol LW (2000). Novel biological processes for the removal of H_2_S and SO_2_ from gas streams. *Environmental Technologies to Treat Sulfur Pollutions; Principles and Engineering*.

[29] van Houten BHGW, Roest K, Tzeneva VA, Dijkman H, Smidt H, Stams AJM (2006). Occurrence of methanogenesis during start-up of a full-scale synthesis gas-fed reactor treating sulfate and metal-rich wastewater. *Water Research*.

[30] Johnson B, Lens PNL, Hulshoff Pol LW (2000). Biological removal of sulfurous compounds from inorganic wastewaters. *Environmental Technologies to Treat Sulfur Pollutions; Principles and Engineering*.

[31] van Houten RT, Hulshoff Pol LW, Lettinga G (1994). Biological sulphate reduction using gas-lift reactors fed with hydrogen and carbon dioxide as energy and carbon source. *Biotechnology and Bioengineering*.

[32] Graboski MS, Herman RG (1984). The production of synthesis gas from methane, coal and biomass. *Catalytic Conversion of Synthesis Gas and Alcohols to Chemicals*.

[33] Sipma J, Henstra AM, Parshina SM, Lens PN, Lettinga G, Stams AJ (2006). Microbial CO conversions with applications in synthesis gas purification and bio-desulfurization. *Critical Reviews in Biotechnology*.

[34] Mörsdorf G, Frunzke K, Gadkari D, Meyer O (1992). Microbial growth on carbon monoxide. *Biodegradation*.

[35] Davidova MN, Tarasova NB, Mukhitova FK, Karpilova IU (1994). Carbon monoxide in metabolism of anaerobic bacteria. *Canadian Journal of Microbiology*.

[36] Sokolova TG, Henstra AM, Sipma J, Parshina SN, Stams AJM, Lebedinsky AV (2009). Diversity and ecophysiological features of thermophilic carboxydotrophic anaerobes. *FEMS Microbiology Ecology*.

[37] Amend JP, Shock EL (2001). Energetics of overall metabolic reactions of thermophilic and hyperthermophilic Archaea and Bacteria. *FEMS Microbiology Reviews*.

[38] Lupton FS, Conrad R, Zeikus JG (1984). CO metabolism of *Desulfovibrio vulgaris* strain Madison: physiological function in the absence or presence of exogeneous substrates. *FEMS Microbiology Letters*.

[39] Jansen K, Thauer RK, Widdel F, Fuchs G (1984). Carbon assimilation pathways in sulfate reducing bacteria. Formate, carbon dioxide, carbon monoxide, and acetate assimilation by *Desulfovibrio baarsii*. *Archives of Microbiology*.

[40] Karpilova II, Davydova MN, Beliaeva MI (1983). The effect of carbon monoxide on the growth of sulfate-reducing bacteria and their oxidation of this substrate. *Nauchnye Doklady Vysshei Shkoly. Biologicheskie Nauki*.

[41] Plugge CM, Balk M, Stams AJM (2002). *Desulfotomaculum thermobenzoicum* subsp. *thermosyntrophicum* subsp. nov., a thermophilic, syntrophic, propionate-oxidizing, spore-forming bacterium. *International Journal of Systematic and Evolutionary Microbiology*.

[42] Stetter KO, Laurer G, Thomm M, Neuner A (1987). Isolation of extremely thermophilic sulfate reducers: evidence for a novel branch of archaebacteria. *Science*.

[43] Stetter KO (1988). *Archaeoglobus fulgidus* gen. nov., sp. nov.: a new taxon of extremely thermophilic archaebacteria. *Systematic and Applied Microbiollogy*.

[44] Svetlichny VA, Sokolova TG, Gerhardt M, Ringpfeil M, Kostrikina NA, Zavarzin GA (1991). *Carboxydothermus hydrogenoformans* gen. nov., sp. nov., a CO-utilizing thermophilic anaerobic bacterium from hydrothermal environments of Kunashir Island. *Systematic and Applied Microbiology*.

[45] Campbell LL, Postgate JR (1965). Classification of the sporeforming sulphate-reducing bacteria. *Bacteriology Reviews*.

[46] Kuever J, Rainey FA, De Vos P, Garrity GM, Jones D, Reiney FA, Schleifer K-H, Whitman WB (2009). Genus *Desulfotomaculum* Campbell and Postgate 1965, 361^AL^. *Bergey’s Manual of Systematic Bacteriology*.

[47] Mori K, Hatsu M, Kimura R, Takamizawa K (2000). Effect of heavy metals on the growth of a methanogen in pure culture and coculture with a sulfate-reducing bacterium. *Journal of Bioscience and Bioengineering*.

[48] Mukhitova FK, Ryazantseva IN, Karpilova II, Belyaeva MI (1983). The utilization of carbon monoxide by bacteria of *Desulfovibrio* genus. *Izevestiya Akademii Nauk SSSR*.

[49] du Preez LA, Odendaal JP, Maree JP, Ponsonby M (1992). Biological removal of sulphate from industrial effluents using producer gas as energy source. *Environmental Technology*.

[50] du Preez LA, Maree JP (1994). Pilot-scale biological sulphate and nitrate removal utilizing producer gas as energy source. *Water Science and Technology*.

[51] van Houten RT, van der Spoel H, van Aelst AC, Hulshoff Pol LW, Lettinga G (1996). Biological sulfate reduction using synthesis gas as energy and carbon source. *Biotechnology and Bioengineering*.

[52] Henstra AM, Sipma J, Rinzema A, Stams AJ (2007). Microbiology of synthesis gas fermentation for biofuel production. *Current Opinion in Biotechnology*.

[53] Perry RH, Green DW, Maloney JO (1997). *Perry’s Chemical Engineers’ Handbook*.

[54] van Houten RT (1996). *Biological sulphate reduction with synthesis gas*.

[55] van Houten RT, Lettinga G, Wijffels RH, Buitelaar RM, Bucke C, Tramper J (1996). Biological sulphate reduction with synthesis gas: microbiology and technology. *Progress in Biotechnology*.

[56] Sipma J, Osuna MB, Parshina SN, Lettinga G, Stams AJM, Lens PNL (2007). H_2_ enrichment from synthesis gas by *Desulfotomaculum* carboxydivorans for potential applications in synthesis gas purification and biodesulfurization. *Applied Microbiology and Biotechnology*.

[57] Min H, Zinder SH (1990). Isolation and characterization of a thermophilic sulfate-reducing bacterium *Desulfotomaculum thermoacetoxidans* sp. nov.. *Archives of Microbiology*.

[58] Henry EA, Devereux R, Maki JS (1994). Characterization of a new thermophilic sulfate-reducing bacterium. *Thermodesulfovibrio yellowstonii*, gen. nov. and sp. nov.: its phylogenetic relationship to *Thermodesulfobacterium* commune and their origins deep within the bacterial domain. *Archives of Microbiology*.

[59] Roberts GP, Youn H, Kerby RL (2004). CO-Sensing mechanisms. *Microbiology and Molecular Biology Reviews*.

[60] King GM, Weber CF (2007). Distribution, diversity and ecology of aerobic CO-oxidizing bacteria. *Nature Reviews Microbiology*.

[61] Oelgeschläger E, Rother M (2008). Carbon monoxide-dependent energy metabolism in anaerobic bacteria and archaea. *Archives of Microbiology*.

[62] Odom JM, Peck HD (1981). Hydrogen cycling as a general mechanism for energy coupling in the sulfate-reducing bacteria. *Desulfovibrio* sp. *FEMS Microbiology Letters*.

[63] Heidelberg JF, Seshadri R, Haveman SA (2004). The genome sequence of the anaerobic, sulfate-reducing bacterium *Desulfovibrio vulgaris* Hildenborough. *Nature Biotechnology*.

[64] Pierik AJ, Hulstein M, Hagen WR, Albracht SPJ (1998). A low-spin iron with CN and CO as intrinsic ligands forms the core of the active site in [Fe]-hydrogenases. *European Journal of Biochemistry*.

[65] Nicolet Y, Piras C, Legrand P, Hatchikian CE, Fontecilla-Camps JC (1999). *Desulfovibrio desulfuricans* iron hydrogenase: the structure shows unusual coordination to an active site Fe binuclear center. *Structure*.

[66] Lemon BJ, Peters JW, Messerschmidt A, Huber R, Poulus T, Wieghardt K (2001). Iron-only hydrogenases. * Handbook of Metalloproteins*.

[67] Greco C, Bruschi M, Heimdal J, Fantucci P, de Gioia L, Ryde U (2007). Structural insights into the active-ready form of [FeFe]-hydrogenase and mechanistic details of its inhibition by carbon monoxide. *Inorganic Chemistry*.

[68] Mityashina SY, Davydova MN (1995). Effects of carbon monoxide on the nucleotide content of *Desulfovibrio desulfuricans* B-1388. *Applied Biochemistry and Microbiology*.

[69] Mityashina SY, Davydova MN (1998). Characteristics of the energy state of *Desulfovibrio desulfuricans* B-1388 cells growing in lactate-sulfate medium under an atmosphere of argon plus carbon monoxide. *Microbiology*.

[70] Davydova M, Sabirova R, Vylegzhanina N, Tarasova N (2004). Carbon monoxide and oxidative stress in *Desulfovibrio desulfuricans* B-1388. *Journal of Biochemical and Molecular Toxicology*.

[71] Lemon BJ, Peters JW (1999). Binding of exogenously added carbon monoxide at the active site of the iron-only hydrogenase (CpI) from *Clostridium pasteurianum*. *Biochemistry*.

[72] Liebgott PP, Leroux F, Burlat B (2010). Relating diffusion along the substrate tunnel and oxygen sensitivity in hydrogenase. *Nature Chemical Biology*.

[73] Chivian D, Brodie EL, Alm EJ (2008). Environmental genomics reveals a single-species ecosystem deep within earth. *Science*.

[74] Shelver D, Kerby RL, He Y, Roberts GP (1997). CooA, a CO-sensing transcription factor from *Rhodospirillum rubrum*, is a CO-binding heme protein. *Proceedings of the National Academy of Sciences of the United States of America*.

[75] Fox JD, Kerby RL, Roberts GP, Ludden PW (1996). Characterization of the CO-induced, CO-tolerant hydrogenase from *Rhodospirillum rubrum* and the gene encoding the large subunit of the enzyme. *Journal of Bacteriology*.

[76] Kerby RL, Ludden PW, Roberts GP (1995). Carbon monoxide-dependent growth of *Rhodospirillum rubrum*. *Journal of Bacteriology*.

[77] Voordouw G (2002). Carbon monoxide cycling by *Desulfovibrio vulgaris* Hildenborough. *Journal of Bacteriology*.

[78] Tarasova NB, Belyaeva MI (1998). The CO dehydrogenase activity of *Desulfovibrio desulfuricans* growing under chemoorganotrophic and chemolithoheterotrophic conditions. *Microbiology*.

[79] Sipma J, Lens PNL, Stams AJM, Lettinga G (2003). Carbon monoxide conversion by anaerobic bioreactor sludges. *FEMS Microbiology Ecology*.

[80] Sipma J, Lettinga G, Stams AJM, Lens PNL (2006). Hydrogenogenic CO conversion in a moderately thermophilic (55^*°*^C) sulfate-fed gas lift reactor: competition for CO-derived H_2_. *Biotechnology Progress*.

[81] Sipma J (2006). *Microbial hydrogenotrophic CO conversions: applications in synthesis gas purification and biodesulfurization*.

[82] Sipma J, Osuna MB, Lettinga G, Stams AJM, Lens PNL (2007). Effect of hydraulic retention time on sulfate reduction in a carbon monoxide fed thermophilic gas lift reactor. *Water Research*.

[83] Vallero MVG, Treviño RHM, Paulo PL, Lettinga G, Lens PNL (2003). Effect of sulfate on methanol degradation in thermophilic (55^*°*^C) methanogenic UASB reactors. *Enzyme and Microbial Technology*.

[84] van Houten RT, Yun SY, Lettinga G (1997). Thermophilic sulphate and sulphite reduction in lab-scale gas-lift reactors using H_2_ and CO_2_ as energy and carbon source. *Biotechnology and Bioengineering*.

[85] Scholten JCM, Conrad R, Stams AJM (2000). Effect of 2-bromo-ethane sulfonate, molybdate and chloroform on acetate consumption by methanogenic and sulfate-reducing populations in freshwater sediment. *FEMS Microbiology Ecology*.

[86] Sipma J, Meulepas RJW, Parshina SN, Stams AJM, Lettinga G, Lens PNL (2004). Effect of carbon monoxide, hydrogen and sulfate on thermophilic (55^*°*^C) hydrogenogenic carbon monoxide conversion in two anaerobic bioreactor sludges. *Applied Microbiology and Biotechnology*.

[87] Oh S-E, van Ginkel S, Logan BE (2003). The relative effectiveness of pH control and heat treatment for enhancing biohydrogen gas production. *Environmental Science and Technology*.

[88] Chen C-C, Lin C-Y, Lin M-C (2002). Acid-base enrichment enhances anaerobic hydrogen production process. *Applied Microbiology and Biotechnology*.

[89] Weijma J, Bots EAA, Tandlinger G, Stams AJM, Hulshoff Pol LW, Lettinga G (2002). Optimisation of sulphate reduction in a methanol-fed thermophilic bioreactor. *Water Research*.

[90] Sparling R, Risbey D, Poggi-Varaldo HM (1997). Hydrogen production from inhibited anaerobic composters. *International Journal of Hydrogen Energy*.

[91] de Smul A, Verstraete W (1999). The phenomenology and the mathematical modeling of the silicone-supported chemical oxidation of aqueous sulfide to elemental sulfur by ferric sulphate. *Journal of Chemical Technology and Biotechnology*.

[92] Cord-Ruwisch R, Seitz H-J, Conrad R (1988). The capacity of hydrogenotrophic anaerobic bacteria to compete for traces of hydrogen depends on the redox potential of the terminal electron acceptor. *Archives of Microbiology*.

